# Association between height and circulating CD34-positive cells taken into account for the influence of enhanced production among elderly Japanese men: a cross-sectional study

**DOI:** 10.18632/aging.101768

**Published:** 2019-01-29

**Authors:** Yuji Shimizu, Hirotomo Yamanashi, Yuko Noguchi, Jun Koyamatsu, Mako Nagayoshi, Kairi Kiyoura, Shoichi Fukui, Mami Tamai, Shin-Ya Kawashiri, Kazuhiko Arima, Takahiro Maeda

**Affiliations:** ^1^Department of Community Medicine, Nagasaki University Graduate School of Biomedical Sciences, Nagasaki, Japan; ^2^Department of Cardiovascular Disease Prevention, Osaka Center for Cancer and Cardiovascular Disease Prevention, Osaka, Japan; ^3^Department of General Medicine, Nagasaki University Hospital, Nagasaki, Japan; ^4^Department of Island and Community Medicine, Nagasaki University Graduate School of Biomedical Sciences, Nagasaki, Japan; ^5^Department of Immunology and Rheumatology, Nagasaki University Graduate School of Biomedical Sciences, Nagsaki, Japan; ^6^Department of Public Health, Nagasaki University Graduate School of Biomedical Sciences, Nagasaki, Japan

**Keywords:** CD34-positive cell, consumptive reduction, elderly men, height, vascular maintenance

## Abstract

Recent studies have revealed an inverse association between height and cardiovascular disease and that endothelial progenitor cells (CD34-positive cells) contribute to vascular maintenance, which is associated with cardiovascular disease. However, evidence of the association between height and CD34-positive positive cells among elderly participants is limited. To assess this association, we conducted a cross-sectional study of 231 elderly Japanese men aged 65–69. Since enhanced production of circulating CD34-positive cells in response to endothelial injury might act have a strong confounding effect on the association between height and circulating CD34-positive cells, the median value for the levels of these cells (0.93 cells/μL) was used to stratify the participants. Multivariable linear regression analysis demonstrated that height was significantly positively associated with circulating CD34-positive cells for those participants with low levels of circulating CD34-positive cells (n=114) but not for those with higher levels (n=117), with a multi-adjusted standardized parameter estimate (β) of 0.27 (p=0.008) for low and 0.11 (0.275) for higher circulating CD34-positive cell levels. The positive association is limited to participants with relatively low circulating CD34-positive cell levels, whose productivity of these cells is not activated. Our findings indicate that height is an indicator of vascular maintenance capability in elderly Japanese men.

## INTRODUCTION

Many recent studies have reported an inverse association between height and cardiovascular disease including stroke incidence [1–4]. In addition, our previous studies indicate that the risk of stroke for participants with short stature could not be explained by the existence of atherosclerosis but could be explained by a lower capability for vascular maintenance [4–9].

On the other hands, CD34-positive cells constitute a known factor that contributes to endothelial repair [10] in conjunction with platelets [11, 12]. Further, the number of circulating CD34-positive cells could indicate capability for endothelial maintenance [13–15]. Therefore, the association between circulating CD34-positive cell levels and height could have an effect on vascular maintenance capability [8, 9].

However, we also reported in a previous study that aggressive endothelial repair not only causes the elevation of circulating CD34-postive cells by increasing the activity of CD34-positive cell production but also causes consumptive reduction of circulating CD34-positive cells [14–17]. Therefore, consumptive reduction following increased production of CD34-positive cell might have a strong confounding effect on the present analysis. Since a high level of CD34-positive cells (≥median value) can be assumed to be associated with endothelial injury [18], limiting the participants of the analysis to those with low levels of CD34-positive cells could reduce the influence of this confounding factor.

To evaluate the impact of height on vascular maintenance capacity for elderly participants, we conducted a cross-sectional study of 231 elderly Japanese men who participated in a general health check-up in 2013–2015.

## RESULTS

No significant correlation between height and age was observed among the present study population, with a simple correlation coefficient of ® = –0.12 (p=0.08). The median circulating CD34-positive cells count for this population was 0.93 cells/μL.

Characteristics of the study population dichotomized according to circulating CD34-positive cell levels based on median values are shown in Table 1. Participants with higher circulating CD34-positive cell levels showed significantly higher white blood cell and platelet concentrations than did those with lower circulating CD34-positive cell levels.

**Table 1:** Characteristics of study population dichotomized by circulating CD34-positive cell levels

	Lower CD34-positive cell levels (<0.93 cells/μL)	Higher CD34-positive cell levels (≥0.93 cells/μL)	p
No. of participants	114	117	
Age, years	67.5 ± 1.3	67.2 ± 1.3	0.189
Systolic blood pressure, mmHg	133 ± 18	133 ± 17	0.694
Diastolic blood pressure, mmHg	79 ± 11	79 ± 11	0.846
Body mass index (BMI), kg/m^2^	22.0 ± 1.7	22.4 ± 1.9	0.078
Serum HDL-cholesterol (HDLc), mg/dL	58 ± 14	58 ± 14	0.860
Serum triglycerides (TG}, mg/dL	112 ± 106	111 ± 57	0.900
Serum γ-glutamyltranspeptidase (γ-GTP), IU/L	43 ± 36	44 ± 44	0.894
Hemoglobin A1c (HbA1c), %	5.7 ± 0.6	5.7 ± 0.5	0.266
Serum creatinine, mg/dL	0.84 ± 0.16	0.83 ± 0.14	0.474
White blood cells, cells/μL	4918 ± 1259	6003 ± 1256	<0.001
Platelets (Plt), ×10^4^/μL	20.5 ± 5.2	23.0 ± 5.8	0.001
Height, cm	164.8 ± 5.9	163.5 ± 5.3	0.096

Values: mean ± standard deviation.

Since platelets constitute a contributing factor to endothelial repair (vascular maintenance) in association with CD34-positive cells [11, 12, 17], and hypertension can be assumed to mask the beneficial effect of circulating CD34-positive cells on endothelial repair [14, 15], we performed a hypertension status stratified analysis to evaluate the production and consumptive reduction of circulating CD34-positive cells. As for non-hypertensive participants, no significant association between platelets and circulating CD34-positive cells was observed for participants with lower circulating CD34-positive cell levels, but significant positive associations were observed for the participants with higher level. As for hypertensive participants, although no significant association between platelets and circulating CD34-positive cells was observed for participants with lower circulating CD34-positive cells level, a significant inverse association was observed for those with higher levels (Table 2).

**Table 2:** Simple correlation coefficients of circulating CD34-positive cells and platelets by hypertension status

	Lower CD34-positive cell levels (<0.93 cells/μL)			Higher CD34-positive cell levels (≥0.93 cells/μL)		
	r		p		r	p
Non-hypertension						
No. of participants		69			74	
Platelets	0.05		0.692	0.36		0.002
Hypertension						
No. of participants		45			43	
Platelets	0.05		0.731	-0.30		0.049

r: correlation coefficient. Circulating CD34-positive cell calculated as logarithmic values.

We previously reported identification of a positive association between height and circulating CD34-positive cells for participants with systolic hypertension but not for those with non-systolic hypertension [8]. However, hypertension is a well-known strong endothelial impairment factor. Since damage to endothelial cells stimulates the production of CD34-positive cells, CD34-positive cell concentration should be elevated in participants with hypertension. On the other hand, aggressive vascular repair (endothelial repair) might cause a consumptive reduction in circulating CD34-positive cell levels since the quantity of those cells can be differentiated into mature cells such as endothelial progenitor cells and foam cells which occur in conjunction with platelets [11, 12]. This explains why consumptive reduction of circulating CD34-positive cells could be observed in participants with higher circulating CD34-positive cell levels but not in those with lower levels. Higher circulating CD34-positive cell levels can therefore be assumed to act as a strong confounding factor on analyses pertaining to associations between height and circulating CD34-positive cells.

**Figure 1:**
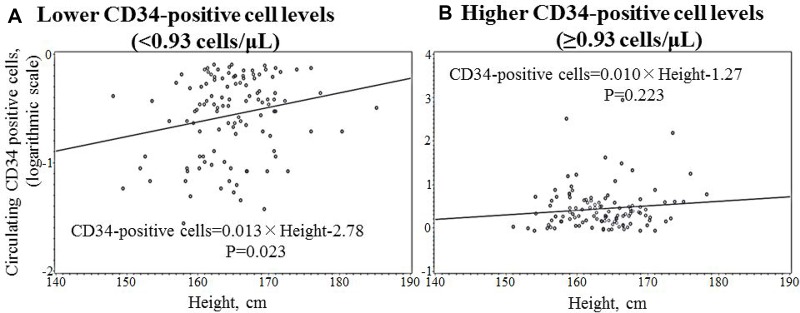
Simple linear regression analysis of circulating CD34-positive cell and height by circulating CD34-positive cell levels.

Simple correlation coefficient (Table 3) and simple linear regression analyses (Figure 1) of associations between circulating CD34-positive cells and height showed significant positive associations only for participants with lower circulating CD34-positive cells levels. Those associations remained unchanged even after adjustments for other possible confounding factors (Table 4).

**Table 3:** Simple correlation coefficients of circulating CD34-positive cell levels and other variables

	Lower CD34-positive cell levels (<0.93 cells/μL)			Higher CD34-positive cell levels (≥0.93 cells/μL)		
	r		p	r		p
No. of participants		114			117	
Age	0.05		0.563	-0.07		0.442
Systolic blood pressure	0.08		0.379	0.13		0.157
Diastolic blood pressure	-0.04		0.656	0.120		0.198
Body mass index (BMI)	0.05		0.634	-0.08		0.370
Serum HDL-cholesterol (HDLc)	-0.02		0.852	0.04		0.659
Serum triglycerides (TG)	-0.14		0.150	0.03		0.708
Serum γ-glutamyltranspeptidase (γ-GTP)	-0.10		0.281	0.14		0.139
Hemoglobin A1c (HbA1c)	-0.05		0.627	-0.03		0.787
Serum creatinine (Cre)	-0.002		0.982	0.11		0.235
White blood cells	0.08		0.426	-0.01		0.894
Height	0.21		0.023	0.11		0.223

r: correlation coefficient. Circulating CD34-positive cell, TG, and γ-GTP calculated as logarithmic values.

**Table 4:** Multiple linear regression analysis of circulating CD34-positive cell levels adjusted with relevant confounding factors

	Lower CD34-positive cell levels (<0.93 cells/μL)			Higher CD34-positive cell levels (≥0.93 cells/μL)		
	Β	β	p	Β	β	p
Participants	114			117		
Age	0.01	0.04	0.691	-0.04	-0.10	0.296
Systolic blood pressure	0.004	0.17	0.109	0.004	0.15	0.134
Body mass index (BMI)	0.009	0.04	0.670	-0.028	-0.11	0.272
Serum HDL-cholesterol (HDLc)	0.0008	0.03	0.781	-0.002	-0.05	0.701
Serum triglycerides (TG)	-0.05	-0.07	0.570	0.03	0.03	0.780
Serum γ-glutamyltranspeptidase (γ-GTP)	-0.08	-0.15	0.222	0.14	0.18	0.084
Hemoglobin A1c (HbA1c)	-0.04	-0.06	0.544	-0.004	-0.004	0.967
Serum creatinine (Cre)	-0.12	-0.05	0.637	0.43	0.13	0.187
White blood cells	0.26×10^-4^	0.09	0.400	-0.41×10^-4^	-0.11	0.314
Height	0.02	0.27	0.008	0.010	0.11	0.275

B: parameter estimate. β: standardized parameter estimate. p: p values for multivariable linear regression models. TG, γ-GTP and circulating CD34-positive cell calculated as logarithmic values.

Further, platelets have been revealed to be closely related to CD34-positive cells during vascular repair [11, 12]. However, even if both platelet production and CD34-positive cell production are stimulated by endothelial injury, aggressive endothelial repair (a cause of atherosclerosis) might result in consumptive reduction of CD34-positive cells but not of platelets because there is a sufficient quantity of platelets [17]. Therefore, the association between platelets and circulating CD34-positive cells could aid evaluation of the influence of stimulated production and consumptive reduction of circulating CD34-positive cells. For our study participants with higher levels of circulating CD34-positive cells, a significant positive association was observed between platelets and circulating CD34-positive cells among those without hypertension, and a significant inverse association among those with hypertension (Table 2). However, those significant associations were not observed among participants with lower levels of circulating CD34-positive cells. These findings partly support the mechanisms mentioned earlier because a significant positive association between platelets and circulating CD34-positive cells indicates an increase in the production of platelets and CD34-positive cells in non-hypertensive participants while a significant inverse association indicates the effect of consumptive reduction of CD34-positive cells in hypertensive participants.

Sensitivity analysis by quartile of circulating CD34-positive cells to detect associations between height and circulating CD34-postive cells showed associations similar to the main results.

A further analysis of participants with lower circulating CD34-positive cells (<0.93 cells/μL), including those with over-nutrition (body mass index (BMI ≥25kg/m^2^), showed that the significant positive associations between height and circulating CD34-positive cells became slightly stronger (Table 5).

**Table 5:** Multiple linear regression analysis of circulating CD34-positive cell levels adjusted with relevant confounding factors among participants including over-nutrition

	Lower CD34-positive cell levels (<0.93 cells/μL)			Higher CD34-positive cell levels (≥0.93 cells/μL)		
	Β	β	p	Β	β	p
Participants	147			183		
Age	0.01	0.02	0.810	-0.02	-0.04	0.573
Systolic blood pressure	0.004	0.20	0.026	0.002	0.05	0.489
Body mass index (BMI)	0.004	0.03	0.771	-0.003	-0.01	0.860
Serum HDL-cholesterol (HDLc)	0.003	0.10	0.308	0.001	0.04	0.688
Serum triglycerides (TG)	0.01	0.02	0.868	0.01	0.01	0.888
Serum γ-glutamyltranspeptidase (γ-GTP)	-0.11	-0.20	0.054	0.10	0.14	0.094
Hemoglobin A1c (HbA1c)	-0.04	-0.07	0.441	0.01	0.01	0.907
Serum creatinine (Cre)	-0.09	-0.04	0.639	-0.01	-0.01	0.877
White blood cells	0.39×10^-4^	0.13	0.139	0.20×10^-4^	0.06	0.501
Height	0.02	0.33	<0.001	0.01	0.14	0.072

B: parameter estimate. β: standardized parameter estimate. p: p values for multivariable linear regression models. TG, γ-GTP and circulating CD34-positive cell calculated as logarithmic values.

To evaluate the production and consumptive reduction of circulating CD34-positive cells in all participants, including those with over-nutrition (BMI≥25kg/m^2^), we also performed a hypertension status stratified analysis for any associations between platelets and circulating CD34-positiv cells. We found associations essentially similar to the main results even though the inverse association which was observed among hypertensive participants with higher circulating CD34-positive cells became of no significant value (Table 6).

**Table 6:** Simple correlation coefficients of circulating CD34-positive cells and platelets by hypertension status among participants including over-nutrition

	Lower CD34-positive cell levels (<0.93 cells/μL)			Higher CD34-positive cell levels (≥0.93 cells/μL)		
	r		p	r		p
Non-hypertension						
No. of participants		79			105	
Platelets	0.04		0.729	0.33		<0.001
Hypertension						
No. of participants		68			78	
Platelets	0.07		0.560	-0.20		0.073

r: Correlation coefficient. Circulating CD34-positive cell levels calculated as logarithmic values.

## DISCUSSION

The main finding of our study of elderly Japanese men is that circulating CD34-positive cell concentration is positively associated with height for participants with lower circulating CD34-positive cell levels but not for participants with higher levels. However, consumptive reduction followed by an increase in production of CD34-positive cells may well mask this positive association between height and or circulating CD34-positive cells for participants with higher circulating CD34-positive cell levels.

Furthermore, in an additional analysis including participants with over-nutrition (BMI≥25kg/m^2^), even an inverse association between platelets and circulating CD34-positive cells was observed for hypertensive participants with higher circulating CD34-positive cell, although the statistical power did not reach significance (Table 6). Since arterial stiffness, which is the result of aggressive vascular repair (endothelial repair), is positively associated with BMI [19–21], participants with over-nutrition (BMI≥25kg/m^2^), which the World Health Organization (WHO) has agreed upon as an international classification of overweight [22], may well have a higher activity of CD34-positive cell production, which is likely to weaken the influence of consumptive reduction. In fact, in our previous studies we reported significant positive associations between circulating CD34-positive cells and BMI [14, 17, 18, 23, 24].

For participants with low levels of circulating CD34-positive cells, we found a significant positive association between height and circulating CD34-positive cells. Bone marrow activity has recently been revealed to be closely associated with vascular maintenance since hematopoietic stem cells (immature cells such as CD34-postive cells) derived from bone marrow reportedly play a major role in vascular homeostasis [10, 25–28]. It is known that hematopoietic bone marrow activity declines with age [29–32], and because height may be positively correlated with total bone marrow volume, it may also influence the age-related decline in hematopoietic bone marrow value, which in turn may exert a crucial influence on the association between height and vascular maintenance capability. Previously, we reported that height is significantly positively associated with hematopoietic capability [9, 33], as evaluated in terms of reticulocyte level, and inversely associated with normocytic normochromic anemia among elderly men [34]. These studies support the hypothetical existence of such a mechanism.

Although our present study employs a small sample size, it is the largest study in the world that deals with circulating CD34-positive cells among the general elderly population who are selected in a strict manner as like previous of our study [8, 9, 33] —subjects were restricted to men in a narrow age and normal BMI range because gender differences, age and high BMI can act as strong confounding factors on the association between height and other variables [35].

Potential limitations of this study warrant consideration. Because creatinine clearance data were not available and estimated glomerular filtration rate (GFR) is not an effective tool for evaluating kidney function for determining the latter’s associations with various body heights [4–7, 33, 34], we were not able to perform an analysis adjusted for exact renal function. However, our study showed that the association between height and circulatory CD34-positive cell remained significant even after adjustment for serum creatinine. Although significant associations exist between height and hematological parameters such as platelet and reticulocyte counts [9, 33, 34], no data was available with regard to the evaluation of endothelial function. Further analyses that include endothelial function-related data such as flow mediated dilation (FMD) will be necessary. Additionally, because this was a cross-sectional study, causal relationships could not be established. However, since height can be regarded as a surrogate marker of childhood social and physical conditions and the target population in the present study was elderly men, we believe this investigation to some extent has the characteristics of a prospective study. Nevertheless, further prospective population-based studies are needed to eliminate the possibility of causal relationships.

In conclusion, circulating CD34-positive cell levels are positively associated with height for participants with relatively low circulating CD34-positive cell levels but not for those with higher levels. Consumptive reduction followed by an increase in production of CD34-positive cells may well mask this positive association between height and circulating CD34-positive cell levels. Our results indicate that height is an indicator of vascular maintenance capacity in elderly Japanese men.

## METHODS

### Study population

To avoid the influence of age on height, this study was comprised of subjects in a narrow age range as like previous studies [8, 9, 33]. The study was conducted during a medical screening program for members of the general population aged 65-69 years who were living in Goto city and Saza town, Nagasaki Prefecture, Japan. A total of 409 Japanese men aged 65–69 years were enrolled after their informed consent had been obtained.

To avoid the influence on the study analyses of inflammatory and hematological diseases, participants with high and low white blood cell counts (≥10,000 cells/μL (n=2) and 1,000 cells/μL< (n=1), respectively) were excluded. Additionally, to avoid the influence of bone marrow-activating medication, participants taking medicine for anemia (n=3) were excluded. Participants lacking evaluable laboratory data (n=59) were also excluded, as were those with low body mass index (BMI) (<18.5kg/m^2^) (n=14) and high BMI (≥25.0kg/m^2^) (n=99) in order to avoid the influence of malnutrition which could be associated with chronic disease and over-nutrition which could be associated with active endothelial repair. As a result, a total of 231 participants were eventually enrolled in the study.

This study was approved by the Ethics Committee for Human Use of Nagasaki University (project registration number: 14051404).

### Data collection and laboratory measurements

Body weight and height were measured with an automatic body composition analyzer (BF-220; Tanita, Tokyo, Japan), after which BMI was calculated. Systolic and diastolic blood pressure were recorded at rest.

Fasting blood samples were collected in a heparin sodium tube, an EDTA-2K tube, a siliconized tube, and a sodium fluoride tube. Fresh samples from the heparin sodium tube were used within 24 hours after collection to determine the number of CD34-positive cells. BD Trucount^TM^ technology (Beckton Dickinson Biosciences, San Jose, CA), an accurate and reproducible single platform assay, endorsed in the International Society of Hematotherapy and Graft Engineering (ISHAGE) guidelines [36] and supported by automated software on the BD FACSCant^TM^ II system, was used to measure the number of circulating CD34-positive cells.

White blood cell and platelet concentrations in samples from the EDTA-2K tube were measured at SRL, Inc. (Tokyo, Japan) with an automated procedure. Serum triglycerides (TG), serum high density lipoprotein cholesterol (HDLc), serum γ-glutamyltranspeptidase (γ-GTP), hemoglobin A1c (HbA_1C_), and serum creatinine were also measured at SRL, Inc. with standard laboratory procedures.

### Statistical analyses

For analysis of circulating CD34-positive cell levels, the participants were stratified into those with higher and lower levels since the influence of the consumptive reduction of circulating CD34-positive cells, which might act as a strong confounding factor on the association between height and circulating CD34-positive cell levels, can weaken in participants with lower levels.

Characteristics of the study population stratified by circulating CD34-positive cell level were expressed as mean ± standard deviation. Circulating CD34-positive-specific simple correlation analysis was used to calculate circulating CD34-positive cell and platelet levels according to hypertension status. we defined hypertension according to previous studies [8, 14, 15, 17], namely, a systolic blood pressure ≥ 140mmHg and/or a diastolic blood pressure ≥ 90mmHg.

A simple correlation analysis and multiple linear regression analysis of circulating CD34-positive cells with relevant factors adjusted for confounding factors based on circulating CD34-positive cell levels were also performed. For the multiple linear regression analysis, adjustments were made for age, systolic blood pressure mmHg), BMI (kg/m^2^), as well as concentrations of HDLc (mg/dL), TG (mg/dL), γ-GTP (IU/L), HbA1c (%), serum creatinine (mg/dL), and white blood cells (cells/μL). Because CD34-positive cell, TG and γ-GTP had a skewed distribution, logarithmic transformation was used for these factors.

For sensitivity analysis, we performed an analysis of the relationship between height and circulating CD34-positive cell by using quartile levels of circulating CD34-positive cells, and we also reused the models but with inclusion of participants with over-nutrition.

All statistical analyses were performed with the SAS system for Windows (version 9.4:); SAS Inc., Cary, NC). All p-values for statistical tests were two-tailed, and values of <0.05 were regarded as statistically significant.

## References

[1] Samaras TT, Elrick H, Storms LH (2004). Is short height really a risk factor for coronary heart disease and stroke mortality? A review. Med Sci Monit.

[2] Honjo K, Iso H, Inoue M, Tsugane S (2011). Adult height and the risk of cardiovascular disease among middle aged men and women in Japan. Eur J Epidemiol.

[3] Park CS, Choi EK, Han KD, Lee HJ, Rhee TM, Lee SR, Cha MJ, Lim WH, Kang SH, Oh S (2018). Association between adult height, myocardial infarction, heart failure, stroke and death: a Korean nationwide population-based study. Int J Epidemiol.

[4] Shimizu Y, Imano H, Ohira T, Kitamura A, Kiyama M, Okada T, Ishikawa Y, Shimamoto T, Yamagishi K, Tanigawa T, Iso H, CIRCS Investigators (2014). Adult height and body mass index in relation to risk of total stroke and its subtypes: the circulatory risk in communities study. J Stroke Cerebrovasc Dis.

[5] Shimizu Y, Nakazato M, Sekita T, Kadota K, Arima K, Yamasaki H, Goto H, Shirahama S, Takamura N, Aoyagi K, Maeda T (2013). Relationship between adult height and body weight and risk of carotid atherosclerosis assessed in terms of carotid intima-media thickness: the Nagasaki Islands study. J Physiol Anthropol.

[6] Shimizu Y, Yoshimine H, Nagayoshi M, Kadota K, Takahashi K, Izumino K, Inoue K, Maeda T (2016). Short stature is an inflammatory disadvantage among middle-aged Japanese men. Environ Health Prev Med.

[7] Shimizu Y, Yoshimine H, Nagayoshi M, Kadota K, Takahashi K, Izumino K, Inoue K, Maeda T (2016). Height correlates with dyslipidemia in non-overweight middle-aged Japanese men. J Physiol Anthropol.

[8] Shimizu Y, Sato S, Koyamatsu J, Yamanashi H, Nagayoshi M, Kadota K, Maeda T (2017). Height is an indicator of vascular maintenance capacity in older men. Geriatr Gerontol Int.

[9] Shimizu Y, Sato S, Koyamatsu J, Yamanashi H, Nagayoshi M, Kadota K, Kawashiri SY, Takahiro Maeda T (2018). Possible mechanism underlying the association between height and vascular remodeling in elderly Japanese men. Oncotarget.

[10] Shi Q, Rafii S, Wu MH, Wijelath ES, Yu C, Ishida A, Fujita Y, Kothari S, Mohle R, Sauvage LR, Moore MA, Storb RF, Hammond WP (1998). Evidence for circulating bone marrow-derived endothelial cells. Blood.

[11] Stellos K, Langer H, Daub K, Schoenberger T, Gauss A, Geisler T, Bigalke B, Mueller I, Schumm M, Schaefer I, Seizer P, Kraemer BF, Siegel-Axel D, May AE, Lindemann S, Gawaz M (2008). Platelet-derived stromal cell-derived factor-1 regulates adhesion and promotes differentiation of human CD34+ cells to endothelial progenitor cells. Circulation.

[12] Daub K, Langer H, Seizer P, Stellos K, May AE, Goyal P, Bigalke B, Schönberger T, Geisler T, Siegel-Axel D, Oostendorp RA, Lindemann S, Gawaz M (2006). Platelets induce differentiation of human CD34+ progenitor cells into foam cells and endothelial cells. FASEB J.

[13] Bielak LF, Horenstein RB, Ryan KA, Sheedy PF, Rumberger JA, Tanner K, Post W, Mitchell BD, Shuldiner AR, Peyser PA (2009). Circulating CD34+ cell count is associated with extent of subclinical atherosclerosis in asymptomatic Amish men, independent of 10-year Framingham risk. Clin Med Cardiol.

[14] Shimizu Y, Sato S, Koyamatsu J, Yamanashi H, Nagayoshi M, Kadota K, Kawashiri SY, Inoue K, Nagata Y, Maeda T (2017). Platelets and circulating CD34-positive cells as an indicator of the activity of the vicious cycle between hypertension and endothelial dysfunction in elderly Japanese men. Atherosclerosis.

[15] Shimizu Y, Sato S, Koyamatsu J, Yamanashi H, Nagayoshi M, Kadota K, Maeda T (2015). Circulating CD34-positive cells, glomerular filtration rate and triglycerides in relation to hypertension. Atherosclerosis.

[16] Shimizu Y, Sato S, Koyamatsu J, Yamanashi H, Nagayoshi M, Kadota K, Kawashiri SY, Maeda T (2017). Possible mechanism underlying the association between higher hemoglobin level and hypertension in older Japanese men. Geriatr Gerontol Int.

[17] Shimizu Y, Sato S, Koyamatsu J, Yamanashi H, Nagayoshi M, Kadota K, Maeda T (2016). Platelets as an indicator of vascular repair in elderly Japanese men. Oncotarget.

[18] Shimizu Y, Sato S, Koyamatsu J, Yamanashi H, Nagayoshi M, Kadota K, Kawashiri SY, Maeda T (2017). Association between high-density lipoprotein-cholesterol and hypertension in relation to circulating CD34-positive cell levels. J Physiol Anthropol.

[19] Rashid SA, Mahmud SA (2015). Correlation between carotid artery intima-media thickness and luminal diameter with body mass index and other cardiovascular risk factors in adults. Sultan Qaboos Univ Med J.

[20] Leite A, Santos A, Monteiro M, Gomes L, Veloso M, Costa M (2012). Impact of overweight and obesity in carotid intima-media thickness of portuguese adolescents. Acta Paediatr.

[21] Ciccone M, Maiorano A, De Pergola G, Minenna A, Giorgino R, Rizzon P (1999). Microcirculatory damage of common carotid artery wall in obese and non obese subjects. Clin Hemorheol Microcirc.

[22] WHO Expert Consultation (2004). Appropriate body-mass index for Asian populations and its implications for policy and intervention strategies. Lancet.

[23] Shimizu Y, Sato S, Koyamatsu J, Yamanashi H, Nagayoshi M, Kawashiri SY, Inoue K, Fukui S, Kondo H, Nakamichi S, Nagata Y, Maeda T (2018). Hepatocyte growth factor and carotid intima-media thickness in relation to circulating CD34-positive cell levels. Environ Health Prev Med.

[24] Shimizu Y, Sato S, Noguchi Y, Koyamatsu J, Yamanashi H, Nagayoshi M, Kadota K, Kawashiri SY, Nagata Y, Maeda T (2017). Triglycerides and blood pressure in relation to circulating CD34-positive cell levels among community-dwelling elderly Japanese men: a cross-sectional study. Environ Health Prev Med.

[25] Takakura N, Watanabe T, Suenobu S, Yamada Y, Noda T, Ito Y, Satake M, Suda T (2000). A role for hematopoietic stem cells in promoting angiogenesis. Cell.

[26] Asahara T, Murohara T, Sullivan A, Silver M, van der Zee R, Li T, Witzenbichler B, Schatteman G, Isner JM (1997). Isolation of putative progenitor endothelial cells for angiogenesis. Science.

[27] Takahashi T, Kalka C, Masuda H, Chen D, Silver M, Kearney M, Magner M, Isner JM, Asahara T (1999). Ischemia- and cytokine-induced mobilization of bone marrow-derived endothelial progenitor cells for neovascularization. Nat Med.

[28] Yamada Y, Takakura N (2006). Physiological pathway of differentiation of hematopoietic stem cell population into mural cells. J Exp Med.

[29] Brusnahan SK, McGuire TR, Jackson JD, Lane JT, Garvin KL, O'Kane BJ, Berger AM, Tuljapurkar SR, Kessinger MA, Sharp JG (2010). Human blood and marrow side population stem cell and Stro-1 positive bone marrow stromal cell numbers decline with age, with an increase in quality of surviving stem cells: correlation with cytokines. Mech Ageing Dev.

[30] Garvin K, Feschuk C, Sharp JG, Berger A (2007). Does the number or quality of pluripotent bone marrow stem cells decrease with age?. Clin Orthop Relat Res.

[31] Guralnik JM, Ershler WB, Schrier SL, Picozzi VJ (2005). Anemia in the elderly: a public health crisis in hematology. Hematology Am Soc Hematol Educ Program.

[32] Cooper B (2011). The origins of bone marrow as the seedbed of our blood: from antiquity to the time of Osler. Proc (Bayl Univ Med Cent).

[33] Shimizu Y, Sato S, Koyamatsu J, Yamanashi H, Nagayoshi M, Kadota K, Maeda T (2016). Height indicates hematopoietic capacity in elderly Japanese men. Aging (Albany NY).

[34] Shimizu Y, Nakazato M, Sekita T, Kadota K, Miura Y, Arima K, Yamasaki H, Goto H, Takamura N, Aoyagi K, Maeda T (2015). Height and drinking status in relation to risk of anemia in rural adult healthy Japanese men: the Nagasaki Islands study. Aging Male.

[35] Asaoka D, Nagahara A, Shimada Y, Matsumoto K, Ueyama H, Matsumoto K, Nakagawa Y, Takeda T, Tanaka I, Sasaki H, Osada T, Hojo M, Watanabe S (2015). Risk factors for osteoporosis in Japan: is it associated with Helicobacter pylori?. Ther Clin Risk Manag.

[36] Sutherland DR, Anderson L, Keeney M, Nayar R, Chin-Yee I (1996). The ISHAGE guidelines for CD34+ cell determination by flow cytometry. International Society of Hematotherapy and Graft Engineering. J Hematother.

